# Bcl-2 Is Involved in Cardiac Hypertrophy through PI3K-Akt Pathway

**DOI:** 10.1155/2021/6615502

**Published:** 2021-03-02

**Authors:** Xianwei Meng, Jun Cui, Guibin He

**Affiliations:** ^1^Department of Cardiovascular Medicine, Luodian Hospital of District, No. 88 Yongshun Road, Shanghai 201908, China; ^2^Shanghai Luodian Town Community Health Service Center, No. 355 Dongtai Road, Shanghai 201908, China

## Abstract

Cardiac hypertrophy (CH) is a common cause of sudden cardiac death and heart failure, resulting in a significant medical burden. The present study is aimed at exploring potential CH-related pathways and the key downstream effectors. The gene expression profile of GSE129090 was obtained from the Gene Expression Omnibus database (GEO), and 1325 differentially expressed genes (DEGs) were identified, including 785 upregulated genes and 540 downregulated genes. Kyoto Encyclopedia of Genes and Genomes (KEGG) and Reactome pathway enrichment analysis of DEGs were then performed. Although there were no pathways enriched by downregulated genes, many CH-related pathways were identified by upregulated genes, including PI3K-Akt signaling pathway, extracellular matrix- (ECM-) receptor interaction, regulation of actin cytoskeleton, and hypertrophic cardiomyopathy (HCM). In the deeper analysis of PI3K-Akt signaling pathway, we found all the signaling transduction pointed to B cell lymphoma-2- (Bcl-2-) mediated cell survival. We then demonstrated that PI3K-Akt signaling pathway was indeed activated in cardiac hypertrophy. Furthermore, no matter LY294002, an inhibitor of the PI3K/AKT signaling pathway, or Venetoclax, a selective Bcl-2 inhibitor, protected against cardiac hypertrophy. In conclusion, these data indicate that Bcl-2 is involved in cardiac hypertrophy as a key downstream effector of PI3K-Akt signaling pathway, suggesting a potential therapeutic target for the clinical management of cardiac hypertrophy.

## 1. Introduction

Cardiac hypertrophy (CH) is the heart's response to stressful situations that impose increased biomechanical stress by increasing muscle mass. This physiological process contributes to reducing the ventricular wall stress, when the heart undergoes a greater than normal workload. Although hypertrophy of the myocardium is a biological response of stress by augmenting cardiac output, prolonged hypertrophy can lead to ventricular arrhythmias, heart failure, and subsequent cardiovascular mortality [1–3]. As reported previously, many signaling transduction pathways were illustrated to contribute to the development of cardiac hypertrophy [4, 5]. Among the CH-related pathways, PI3K-Akt signaling pathway, which is activated by many types of cellular stimuli or toxic insults to regulate fundamental cellular processes including protein synthesis, proliferation, and survival, was well established [6]. It is demonstrated that sustained activated PI3K in the heart aggravates cardiac hypertrophy and myocardial dysfunction; once PI3K is completely blocked, the hearts lose the hypertrophic response to physiological stimuli [7]. The PI3K-promoting hypertrophic response needs the involvement of PI3K downstream AKT [8], GSK3*β* [9], mTOR [10], P70S6K, and eIF-4E [11], which are involved in the regulation of fundamental cellular processes, including metabolism, glucose uptake, proliferation, and protein synthesis. Furthermore, we found that all the fundamental cellular processes were assigned towards a single goal of cell survival. However, the central downstream effector of the PI3K-Akt signaling pathway, which regulates cell survival, remains incompletely defined.

Bcl-2 is a founding member of the BCL-2 apoptosis regulatory protein family, which can induce (proapoptotic) or inhibit (antiapoptotic) apoptosis [12]. Bcl-2 is overexpressed in more than half of human cancers [13]. As an important oncogene, Bcl-2 can inhibit cell apoptosis by inhibiting the activation of apoptosis proteins (such as BAX and BAK), thereby promoting the survival of cancer cells [14]. The expression of Bcl-2 in human breast cancer is associated with a good prognosis, and ongoing studies have shown that destroying Bcl-2 can cause cell death [15]. Bcl-2 and related cytoplasmic proteins are key regulators of apoptosis, and the cell suicide program is essential for the development, tissue homeostasis, and protection of pathogens. Previous reports indicate that whether cells should survive or die depends largely on the Bcl-2 family of antiapoptotic and apoptosis regulators [16]. Bcl-2 has a cell cycle inhibitory function, which can be separated from improving cell survival [17].

In this study, we reanalyzed the gene expression profile of GSE129090 to explore the molecular mechanism of cardiac hypertrophy. KEGG enrichment analysis showed that the upregulated DEGs were enriched in PI3K-Akt signaling pathway and the central downstream effector was Bcl-2, which functioned in cell survival. To verify the results above, the validation experiments were performed. It was demonstrated that the cross-sectional area in the TAC group was significantly higher than that in the Sham group, accompanying the activation of PI3K-Akt signaling pathway, resulting from the level of phosphorylated PI3K, AKT, GSK3*β*, mTOR, P70S6K, and eIF-4E. Once the phosphorylation of PI3K or Bcl-2 was blocking by their selective inhibitors, the development of CH was retarded. In conclusion, these data indicate that Bcl-2 is involved in cardiac hypertrophy as a central downstream effector of PI3K-Akt signaling pathway, suggesting a potential therapeutic target for the clinical management of cardiac hypertrophy.

## 2. Methods and Materials

### 2.1. Data Collection

The gene expression profile of GSE129090 was obtained from the Gene Expression Omnibus (GEO) database (http://www.ncbi.nlm.nih.gov/geo/) based on the platform of Illumina MouseRef-8 v2.0 expression beadchip. A total of 6 samples were available, including 3 hypertrophic cardioymocytes (hypertrophy induced by trans-aortic constriction (TAC)) samples and 3 normal adult cardiomyocyte (ACM) samples.

### 2.2. Identification for DEGs

For the analysis of DEGs, the expression data were first normalized using the normalizeBetweenArray function from R package “Limma” [18]. Then, the normalized data were used to analyze DEGs using the Limma software package in the R software (http://www.bioconductor.org/packages/release/bioc/html/limma.html) [19]. The cut-off value was set as *P* value <0.05 and log_2_(∣fold change (FC)∣) > 1 for DEG analysis [20].

### 2.3. Pathway Analysis of DEGs

The Kyoto Encyclopedia of Genes and Genomes (KEGG: http://www.genome.ad.jp/KEGG) and Reactome (https://reactome.org/) pathway enrichment analysis were performed to investigate the signaling pathways that were related to the unique DEGs.

### 2.4. Animals and Transverse Aortic Constriction (TAC) Model

Male C57BL/6J mice aged 10 weeks were purchased from Shanghai SLAC Laboratory Animal CO, LTD (China). The experimental procedures were approved by the Institutional Animal Ethical Committee of Luodian hospital of Baoshan District. The experiments were proceeded according to NIH guidelines for Care and Use of Laboratory Animals (NIH, 8th Edition, 2011). The mice were maintained in individually ventilated cages (at 22°C, 12 h light/dark cycle) with free access to standard laboratory chow. 40 rats were randomly divided into 4 groups (10 for each group), which were, namely, Sham group, TAC group, TAC+ LY294002 group, and TAC + Venetoclax group.

TAC operation was performed as a previous report [21]. Briefly, for the TAC group, after carotid arteries explosion and 60%-75% diameter of ligation, mice were administrated with normal saline (NS, 1 ml/day) for 8 weeks; for the TAC + LY294002 group, mice were treated with LY294002 (20 mg/kg/day, Sigma, USA) for 8 weeks by intraperitoneal injections; for the TAC+ Venetoclax group, mice were treated with Venetoclax (100 mg/kg/day, Selleckchem) for 8 weeks by oral gavage.

After administration of TAC operation, mice were maintained for another 8 weeks. Subsequently, the hearts were collected and fixed with 10% neutral formalin and preserved in -80°C for further research.

### 2.5. Western Blotting Analysis

Standard western blot analysis was performed as described in the literature [22]. The primary antibodies used in this study were anti-AKT (ab32505, Abcam), anti-phospho-AKT (ab81283, Abcam), anti-GSK3*β* (ab32391, Abcam), anti-phospho-GSK3*β* (ab131097, Abcam), anti-mTOR (ab2732, Abcam), anti-phospho-mTOR (ab84400, Abcam), anti-eIF-4E (ab32024, Abcam), anti-phosphoeIF-4E (ab76256, Abcam), anti-PI3K (#4257, CST), anti-phospho-PI3K (#4228, CST), anti-P70S6K (#2708, CST), anti-phospho-P70S6K (#9208, CST), and anti-Actin (ab8227, Abcam).

### 2.6. Quantitative Real-Time PCR

Total RNA was isolated from heart tissues using TRIzol Reagent (Invitrogen), and the qRT-PCR experiments were performed according to the literature [22] using SYBR Green (Roche). The expression levels of atrial natriuretic peptide (ANP), brain natriuretic peptide (BNP), and *β*-myosin heavy chain (*β*-MHC) were used as markers of cardiac hypertrophy [23]. GAPDH was used as control, and the 2^−*ΔΔ*Ct^ method was used to measure the relative gene expressions. All the primers used in this study were obtained from Sangon (Shanghai, China). The real-time PCR primers were as follows:

ANP-forward: 5′-ACCTGCTAGACCACCTGGAG-3′, ANP-reverse: 5′-CCTTGGCTGTTATCTTCGGTACCGG-3′; BNP-forward: 5′-GAGGTCACTCCTATCCTCTGG-3′, BNP-reverse: 5′-GCCATTTCCTCCGACTTTTCTC-3′; *β*-MHC-forward: 5′-CCGAGTCCCAGGTCAACAA-3′, *β*-MHC-reverse: 5′-CTTCACGGGCACCCTTGGA-3′; GAPDH-forward: 5′-TTGCTTCAGGGTTTCATCCAG-3′, GAPDH-reverse: 5′-GACACTCGCTCAGCTTCTTG-3′.

### 2.7. Histological Analysis

8 weeks after TAC or sham surgery, the animals were sacrificed, and the hearts were arrested with a 10% potassium chloride solution at end-diastole and then fixed in 10% formalin. The paraffin-embedded hearts were cut transversely into 4-5 *μ*m sections. Heart sections were stained with HE, and the cell size was measured using a quantitative digital image analysis system (ImageJ, 1.52a).

### 2.8. Statistical Analysis

All experiments were performed in three independent parallel experiments, and the results were shown as mean ± standard deviation (SD). Differences between groups were estimated using unpaired Student's *t*-test. A two-tailed value of *P* valve <0.05 was considered statistically significant.

## 3. Results

### 3.1. Identification of DEGs and Enrichment Pathway Analyses

**Figure 1:**
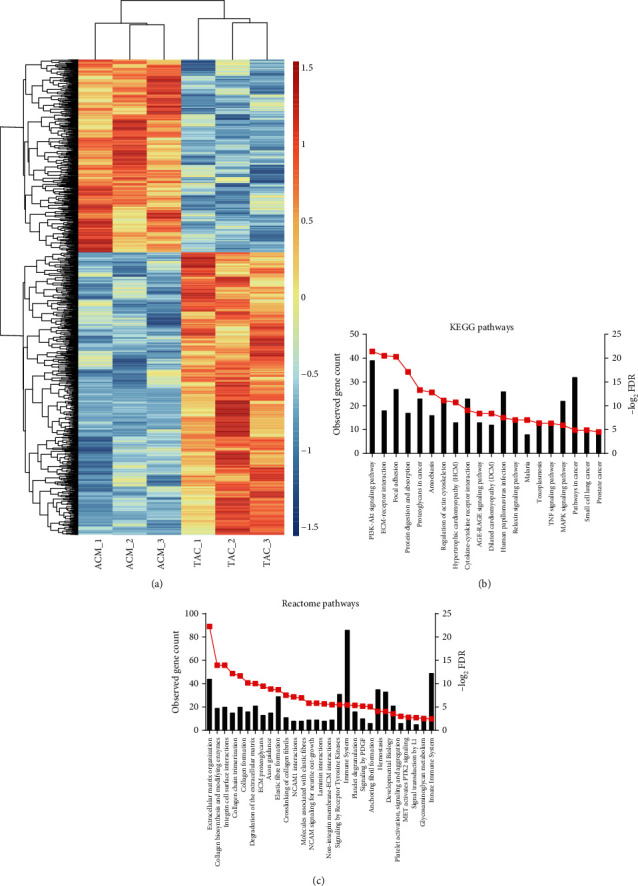
Selection of DEGs and function annotation: (a) heat map of DEGs (785 upregulated and 540 downregulated genes); (b) KEGG pathway analysis of upregulated DEGs; (c) Reactome pathway analysis of upregulated DEGs.

The study included 3 TAC samples and 3 ACM samples. A total of 1325 DEGs were identified after the analysis of GSE129090 by Limma package in R language. Of these, 785 were upregulated, and 540 were downregulated in TAC samples compared with ACM samples. A heat map of DEGs was shown in Figure 1(a).

For a deeper insight into the DEGs, we performed KEGG and Reactome pathway enrichment analyses. Upregulated DEGs were mainly enriched in CH-related pathways (Figure 1(b) and 1(c)), such as PI3K-Akt signaling pathway, ECM-receptor interaction, regulation of actin cytoskeleton, hypertrophic cardiomyopathy (HCM), and cytokine-cytokine receptor interactions. However, the downregulated DEGs were not significantly enriched in any pathway.

### 3.2. PI3K-Akt Signaling Pathway in CH Development

**Figure 2:**
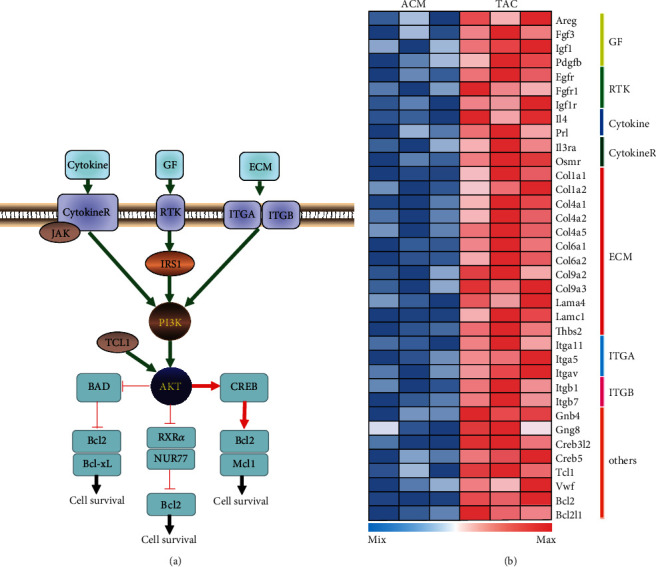
PI3K-Akt signaling pathway in CH: (a) PI3K-Akt signaling pathway in CH manually curated based on KEGG; (b) heat map of DEGs in PI3K-Akt signaling pathway. GF: growth factors; RTK: receptor tyrosine kinase; ECM: extracellular matrix; ITGA: integrin subunit alpha; ITGB: integrin subunit beta; CytokineR: cytokine receptor.

Given that the upregulated DEGs were most significantly enriched in PI3K-Akt signaling pathway, we selected the 36-related genes for further study (Figure 2(a)). Among the 36 selected genes, 28 genes were involved in ligand-receptor interaction, 18 of which were ECM-ITGA/B interaction (Figure 2(b)), showing that the development of CH was mainly promoted by the microenvironment. More importantly, we found that all the signaling transduction pointed to Bcl-2-mediated cell survival. These results prompted us to hypothesize that PI3K-Akt signaling pathway-induced Bcl-2-mediated cell survival was involved in the development of CH.

### 3.3. PI3K-Akt Signaling Pathway Was Involved in the Response to Hypertrophic Stimuli

**Figure 3:**
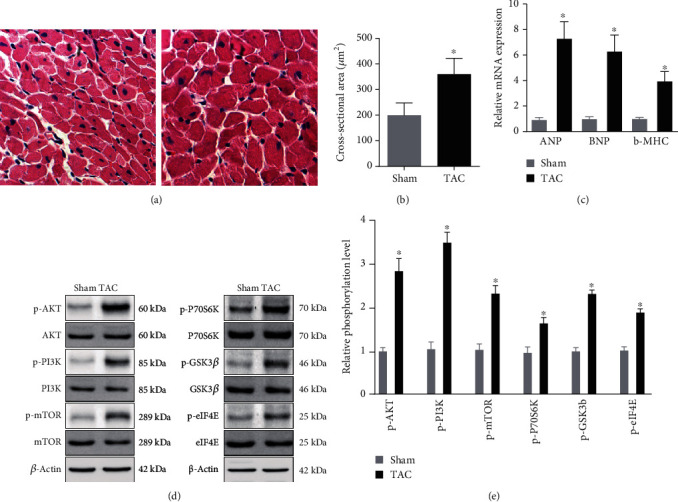
PI3K-Akt signaling pathway was involved in the response to hypertrophic stimuli: (a) histological analyses of the hematoxylin and eosin (H&E) staining of each group of mice at 8 weeks after TAC or sham surgery (*n* = 10); (b) statistical results for the cardiomyocyte cross-sectional area (CSA, *n* = 50 cells); (c) real-time PCR analysis of the hypertrophy markers atrial natriuretic peptide (ANP), brain natriuretic peptide (BNP), and *β*-myosin heavy chain (*β*-MHC); (d) the levels of total and phosphorylated AKT, GSK3*β*, mTOR, PI3K, P70S6K, and eIF-4E expression in heart tissues of mice in the indicated groups; (e) quantitative results of (d) (*n* = 10). ^∗^*P* < 0.05 versus Sham group.

Given that PI3K-Akt signaling pathway was involved in the development of cardiac hypertrophy, we first examined whether PI3K-Akt signaling pathway was activated in cardiac hypertrophy tissues. As shown in Figures 3(a)–3(c), we successfully constructed the TAC mice model. The cross-sectional area (CSA) was significantly higher in the TAC group than that in the Sham group, and the expression of ANP, BNP, and *β*-MHC was increased in the TAC group. As expected, it was observed that PI3K, AKT, GSK3*β*, mTOR, P70S6K, and eIF-4E were significantly phosphorylated in the TAC group, suggesting that PI3K-Akt signaling pathway was indeed activated in cardiac hypertrophy (Figures 3(d) and 3(e)).

### 3.4. LY294002 And Venetoclax Protected against Cardiac Hypertrophy

**Figure 4:**
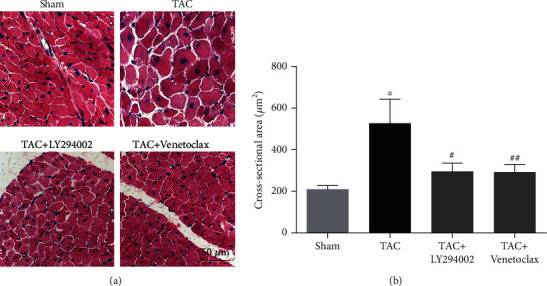
LY294002 and Venetoclax protected against cardiac hypertrophy: (a) histological analyses of the hematoxylin and eosin (H&E) staining; (b) statistical results for the cardiomyocyte cross-sectional area (CSA, *n* = 50 cells). ^∗^*P* < 0.05 versus Sham group. #*P* < 0.05 versus TAC group.

To further examine whether PI3K-Akt signaling pathway had a causative role in the development of cardiac hypertrophy and Bcl-2 was the central downstream effector, additional in vivo experiments were performed. We treated the TAC mice with LY294002 (a PI3K inhibitor that prevents PI3K phosphorylation) or Venetoclax (a selective Bcl-2 inhibitor) as described in Methods and Materials. We found that no matter LY294002 or Venetoclax protected against TAC-induced cardiac hypertrophy (Figure 4(a)). The cross-sectional area was decreased in the treatment group. The results determined that PI3K-Akt signaling pathway played a critical role in TAC-induced CH, and Bcl-2-mediated cell survival was the key cause of the cardiovascular lesions.

## 4. Discussion

Cardiac hypertrophy is one of the most common causes of heart failure, which is increasing in prevalence and is a debilitating disease with high rates of mortality and morbidity worldwide [24]. Although many studies have reported that long-term cardiac hypertrophy increases the likelihood of heart failure, treatment of cardiac hypertrophy has not been well defined, due to the obscure molecular mechanism [25]. Recently, multiple CH-related signaling pathways have been identified in many individual studies, such as the PI3K-Akt, calcineurin/NFAT, and MAPK pathway [26]. Originally, it was thought that the pharmacological agents that selectively modulate the CH-related pathways could inhibit the development of pathological cardiac hypertrophy, but up to now, no effective drugs targeting cardiac hypertrophy have been found [27]. The reason is clear that it is impossible to inhibit all the pathways relating to cardiac hypertrophy. So, finding the central downstream effectors of the CH-related pathways has attracted our attention. In this study, we reanalyzed the gene expression profile of GEO129090, including 3 TAC mice samples and 3 ACM mice samples. 1325 DEGs were identified using R, including 785 upregulated genes and 540 downregulated genes. KEGG and Reactome pathway enrichment analysis of DEGs were then performed. Although there were no pathways identified by downregulated genes, many CH-related pathways were identified by upregulated genes, including PI3K-Akt signaling pathway, ECM-receptor interaction, regulation of actin cytoskeleton, and hypertrophic cardiomyopathy (HCM). The genes involved in the ECM-receptor interaction signaling pathway found in ductal breast carcinoma could be used as effective independent prognostic biomarkers for ductal breast carcinoma. The reorganization of the actin cytoskeleton was a key mechanical driving force for cancer cells to acquire invasive properties. Related genes regulated cell shape by affecting the actin cytoskeleton and were important regulators of migration and proliferation. HCM is the most common genetic heart disease in humans and causes significant morbidity and mortality [28].

In the deeper analysis of PI3K-Akt signaling pathway, we found all the signaling transduction pointed to Bcl-2-mediated cell survival. These results showed that a microenvironment-promoted, PI3K-Akt signaling pathway-activated, and Bcl-2-mediated cell survival may be the key cause of cardiac hypertrophy. To verify the results from the bioinformatical analysis, we then examined the PI3K-Akt signaling pathway in TAC-induced cardiac hypertrophy samples. As reported previously, it was observed that PI3K, AKT, GSK3*β*, mTOR, P70S6K, and eIF-4E were significantly phosphorylated in TAC mice, suggesting that PI3K-Akt signaling pathway was indeed activated in cardiac hypertrophy.

Cardiac hypertrophy is the adaptive response of the heart muscle to pressure or volume overload [29]. PI3K is a highly conserved lipid kinase involved in physiological cardiac hypertrophy (PHH) [30]. The AKT pathway is an important intracellular signaling pathway in eukaryotic cells, especially a regulator of cardiac hypertrophy [31]. There is increasing evidence proving that autophagy is involved in the regulation of cardiac hypertrophy [32]. Studies have shown that related genes can inhibit cardiac hypertrophy caused by pressure overload by inhibiting the AKT/mTOR pathway to promote autophagy [33]. The role of PI3K/Akt/mTOR pathway in cardiac hypertrophy has been fully demonstrated. Dioscin improves cardiac hypertrophy by inhibiting the Akt/GSK3*β*/mTOR pathway [34]. Studies have shown that Apelin-13 promotes cardiomyocyte hypertrophy through PI3K-Akt-ERK1/2-p70S6K and PI3K-induced autophagy [35]. Phosphorylation of eIF-4E is a mechanism by which increased heart load is related to the accelerated rate of protein synthesis [36]. As we know, all the examined proteins, PI3K, AKT, GSK3*β*, mTOR, p70S6K, and eIF-4E, are regulators but not effectors. So, we hypnotized that the downstream protein of PI3K-Akt signaling pathway, Bcl-2, was the effector. In our study of TAC mice treated with inhibitors in vivo, we found that no matter LY294002, an inhibitor of the PI3K/AKT signaling pathway, or Venetoclax, a selective Bcl-2 inhibitor, protected against cardiac hypertrophy. These results suggested that PI3K-AKT signaling pathway was involved in cardiac hypertrophy by regulating Bcl-2-mediated cell survival.

In conclusion, the present study evidences that Bcl-2 works as the central downstream effector of PI3K-Akt signaling pathway to keep cardiomyocyte survival, resulting in cardiac hypertrophy. It is indicated that Bcl-2 is of great possibility to be a therapeutic target for the clinical management of cardiac hypertrophy. This study provides novel and useful information for the potential functions of Bcl-2 and at the same time provides a new direction for the study of the mechanism of CH.

## Data Availability

Please contact the corresponding author for all data requests.

## References

[1] Oldfield C. J., Duhamel T. A., Dhalla N. S. (2019). Mechanisms for the transition from physiological to pathological cardiac hypertrophy. *Canadian Journal of Physiology and Pharmacology*.

[2] Tamargo J., Lopez-Sendon J. (2011). Novel therapeutic targets for the treatment of heart failure. *Nature Reviews. Drug Discovery*.

[3] Koitabashi N., Kass D. A. (2011). Reverse remodeling in heart failure--mechanisms and therapeutic opportunities. *Nature Reviews. Cardiology*.

[4] Heineke J., Molkentin J. D. (2006). Regulation of cardiac hypertrophy by intracellular signalling pathways. *Nature Reviews. Molecular Cell Biology*.

[5] Lorell B. H., Carabello B. A. (2000). Left ventricular hypertrophy: pathogenesis, detection, and prognosis. *Circulation*.

[6] Guan P., Sun Z. M., Wang N. (2019). Resveratrol prevents chronic intermittent hypoxia-induced cardiac hypertrophy by targeting the PI3K/AKT/mTOR pathway. *Life Sciences*.

[7] Aoyagi T., Matsui T. (2011). Phosphoinositide-3 kinase signaling in cardiac hypertrophy and heart failure. *Current Pharmaceutical Design*.

[8] DeBosch B., Sambandam N., Weinheimer C., Courtois M., Muslin A. J. (2006). Akt2 regulates cardiac metabolism and cardiomyocyte survival. *The Journal of Biological Chemistry*.

[9] Sugden P. H., Fuller S. J., Weiss S. C., Clerk A. (2008). Glycogen synthase kinase 3 (GSK3) in the heart: a point of integration in hypertrophic signalling and a therapeutic target? A critical analysis. *British Journal of Pharmacology*.

[10] Clemente C. F., Xavier-Neto J., Dalla Costa A. P. (2012). Focal adhesion kinase governs cardiac concentric hypertrophic growth by activating the AKT and mTOR pathways. *Journal of Molecular and Cellular Cardiology*.

[11] Yang Z., Ming X. F. (2012). mTOR signalling: the molecular interface connecting metabolic stress, aging and cardiovascular diseases. *Obesity Reviews*.

[12] Gross A., McDonnell J. M., Korsmeyer S. J. (1999). BCL-2 family members and the mitochondria in apoptosis. *Genes & Development*.

[13] Nahta R., Esteva F. J. (2003). Bcl-2 antisense oligonucleotides: a potential novel strategy for the treatment of breast cancer. *Seminars in Oncology*.

[14] Youle R. J., Strasser A. (2008). The BCL-2 protein family: opposing activities that mediate cell death. *Nature Reviews. Molecular Cell Biology*.

[15] Del Bufalo D., Biroccio A., Leonetti C., Zupi G. (1997). Bcl-2 overexpression enhances the metastatic potential of a human breast cancer line. *The FASEB Journal*.

[16] Czabotar P. E., Lessene G., Strasser A., Adams J. M. (2014). Control of apoptosis by the BCL-2 protein family: implications for physiology and therapy. *Nature Reviews. Molecular Cell Biology*.

[17] Vairo G., Innes K. M., Adams J. M. (1996). Bcl-2 has a cell cycle inhibitory function separable from its enhancement of cell survival. *Oncogene*.

[18] Smyth G. K., Michaud J., Scott H. S. (2005). Use of within-array replicate spots for assessing differential expression in microarray experiments. *Bioinformatics*.

[19] Kerr M. K. (2003). Linear models for microarray data analysis: hidden similarities and differences. *Journal of Computational Biology*.

[20] Li J. Y., Zheng L. L., Wang T. T., Hu M. (2016). Functional annotation of metastasis-associated microRNAs of melanoma: a meta-analysis of expression profiles. *Chinese Medical Journal*.

[21] de Almeida A. C., van Oort R. J., Wehrens X. H. (2010). Transverse aortic constriction in mice. *JoVE (Journal of Visualized Experiments)*.

[22] Li H., Hong J., Wijayakulathilaka W. (2019). Long non-coding RNA SNHG4 promotes cervical cancer progression through regulating c-Met via targeting miR-148a-3p. *Cell Cycle*.

[23] Bao Q., Chen L., Li J. (2017). Role of microRNA-124 in cardiomyocyte hypertrophy inducedby angiotensin II. *Cellular and Molecular Biology (Noisy-le-Grand, France)*.

[24] Rosca M. G., Tandler B., Hoppel C. L. (2013). Mitochondria in cardiac hypertrophy and heart failure. *Journal of Molecular and Cellular Cardiology*.

[25] Sugden P. H., Clerk A. (1998). Cellular mechanisms of cardiac hypertrophy. *Journal of Molecular Medicine (Berlin, Germany)*.

[26] Asokan Shibu M., Kuo W. W., Kuo C. H. (2017). Potential phytoestrogen alternatives exert cardio-protective mechanisms via estrogen receptors. *Biomedicine*.

[27] Krystof V., Chamrád I., Jorda R., Kohoutek J. (2010). Pharmacological targeting of CDK9 in cardiac hypertrophy. *Medicinal Research Reviews*.

[28] Marian A. J., Roberts R. (2001). The molecular genetic basis for hypertrophic cardiomyopathy. *Journal of Molecular and Cellular Cardiology*.

[29] Takeda N., Manabe I., Uchino Y. (2010). Cardiac fibroblasts are essential for the adaptive response of the murine heart to pressure overload. *The Journal of Clinical Investigation*.

[30] McMullen J. R., Izumo S. (2006). Role of the insulin-like growth factor 1 (IGF1)/phosphoinositide-3-kinase (PI3K) pathway mediating physiological cardiac hypertrophy. *Novartis Foundation symposium*.

[31] Li J., Yuan Y. P., Xu S. C. (2017). Arctiin protects against cardiac hypertrophy through inhibiting MAPKs and AKT signaling pathways. *Journal of Pharmacological Sciences*.

[32] Xue R., Jiang J., Dong B. (2017). DJ-1 activates autophagy in the repression of cardiac hypertrophy. *Archives of Biochemistry and Biophysics*.

[33] Zhao D., Wang W., Wang H. (2017). PKD knockdown inhibits pressure overload-induced cardiac hypertrophy by promoting autophagy via AKT/mTOR pathway. *International Journal of Biological Sciences*.

[34] Chen L., Li Q., Lei L., Li T. (2018). Dioscin ameliorates cardiac hypertrophy through inhibition of the MAPK and Akt/GSK3*β*/mTOR pathways. *Life Sciences*.

[35] Xie F., Liu W., Feng F. (2015). Apelin-13 promotes cardiomyocyte hypertrophy via PI3K-Akt-ERK1/2-p70S6K and PI3K-induced autophagy. *Acta biochimica et biophysica Sinica*.

[36] Wada H., Ivester C. T., Carabello B. A., Cooper G., McDermott P. J. (1996). Translational initiation factor eIF-4E. *The Journal of Biological Chemistry*.

